# Histone Demethylase AMX-1 Regulates Fertility in a p53/CEP-1 Dependent Manner

**DOI:** 10.3389/fgene.2022.929716

**Published:** 2022-06-30

**Authors:** Xiaojing Ren, Sisi Tian, Qinghao Meng, Hyun-Min Kim

**Affiliations:** ^1^ School of Pharmaceutical Science and Technology, Tianjin University, Tianjin, China; ^2^ Division of Natural and Applied Sciences, Duke Kunshan University, Kunshan, China

**Keywords:** LSD2, histone methylation, sterility, fertility, Piwi, transposon, p53, CEP-1

## Abstract

Histone methylation shapes the epigenetic configuration and adjusts multiple fundamental nuclear processes, including transcription, cell cycle control and DNA repair. The absence of histone demethylase LSD1/SPR-5 leads to progressive fertility defects as well as a reduction in brood size. Similarly, *C. elegans* LSD2 homolog AMX-1 has been implicated in regulating H3K4me2 and maintaining interstrand crosslinks (ICL) susceptibility. However, the mechanisms of how lack of AMX-1 induces sterility have not been addressed so far. This study investigated the histone demethylase AMX-1 in *C. elegans* and uncovered how *amx-1* contributes to sterility in a p53/CEP-1 dependent manner. We show that while sterility in *spr-5* mutants exhibited progressive over generations, *amx-1* mutants displayed non-transgenerational fertility defects. Also, *amx-1* mutants exhibited a reduced number of sperms and produced low brood size (LBS) or sterile worms that retain neither sperms nor germline nuclei, suggesting that fertility defects originated from germline development failure. Surprisingly, sterility exhibited in *amx-1* was mediated by p53/CEP-1 function. Consistent with this result, upregulation of Piwi expression in *amx-1* mutants suggested that AMX-1 is essential for germline development by regulating Piwi gene expressions. We propose that AMX-1 is required for proper Piwi expression and transposon silencing in a p53/CEP-1 dependent manner; thus, the absence of AMX-1 expression leads to defective meiotic development and sterility. This study elucidates how LSD2/AMX-1 contributes to sterility, therefore, expanding the boundaries of histone demethylase function.

## Introduction

Since Waddington first proposed the concept of epigenetics in 1942, epigenetic studies have been investigated to identify how gene expression altered without modifying the nucleotide sequences (Goldberg et al., 2007; Allis and Jenuwein, 2016). Classically, the term Epigenetics is restricted to the heritable changes in gene expression that do not modify the nucleotide sequence. However, there is controversy in the field as some employ the term epigenetics to refer to differences in gene expression regardless of the heritability and others use it to refer to transgenerational effects and/or inherited expression states. So far, multiple types of epigenetic marks have been discovered including methylation, acetylation, phosphorylation, ubiquitination and sumoylation, although not all of them have been shown to be heritable.

Chromatin is the complex made up of DNA, histones and associated proteins responsible for packaging DNA molecules into the nucleus (Kornberg and Lorch, 1999; Neganova et al., 2020). Histone modifications, including acetylation and methylation, alter chromatin structure and influence gene expression in an epigenetic manner. Histone demethylases can be broadly classified into two families based on their enzymatic action: amine oxidase demethylases (LSD1/KDM1A and LSD2/KDM1B) and Jumonji C domain (JmjC) family (Nottke et al., 2009; Swahari and West, 2019).

Histone demethylase LSD1 has been related to numerous biological processes. For examples, studies have shown that lysine-specific histone demethylase LSD1 is required for proper germline maturation, DNA damage repair, chromosome segregation and aneuploidy (Nottke et al., 2011; Adhikari and Liu, 2014; Kim et al., 2015; Greer et al., 2016; Kim et al., 2018; Liu et al., 2020). Similarly, In *C. elegans* study, a lack of a mammalian LSD1 homolog SPR-5 exhibits inherited accumulation of the euchromatic H3K4me2 methylation mark along with a progressive decline in fertility (Katz et al., 2009). Defective fertility becomes severer with generations, resulting in a sterile phenotype after multiple generations. In addition, *spr-5* mutants affect the lifespan of *C. elegans* and double-strand breaks repair (McColl et al., 2008; Nottke et al., 2011; Greer et al., 2016). Lastly, SPR-5 coordinate with FANCM/FNCM-1 to cope with replication stress and maintain H3K4me2 level (Kim et al., 2018). These observations suggest histone demethylase’s multiple roles and not restricted in maintaining H3H4me2 level.

*C. elegans* has three homologs of mammalian LSD1/2 histone demethylase: LSD1 homologs SPR-5 and LSD-1 and LSD2 homolog AMX-1. While most studies focused on LSD1 homolog SPR-5, the function of the LSD2 homolog AMX-1 was not very well understood until recently. Since all three histone demethylases belong to LSD1/2 homologs, which are involved in chromatin remodeling and modulating gene expressions, it has been considered that they may perform similar and redundant functions, thus compensating one another. Consistent with this idea, both AMX-1 and SPR-5 are implicated in fertility defect, sterility and lifespan (Katz et al., 2009; Greer et al., 2016; Zhang et al., 2021), thus connecting fertility with histone methylation status. However, since SPR-5 and AMX-1 are implicated in DNA double-strand break repair (DSBR) and Interstrand Crosslink Repair (ICLR), respectively (Wang et al., 2020; Zhang et al., 2021), we postulate that phenotypes observed in two histone demethylases might originate from separate pathways.

Until now, systematic analysis of how *amx-1, per se,* contributes to defective fertility or sterility has not been investigated. Given that AMX-1 is requisite for proper fertility, we examined the mechanism of how AMX-1 contributes to fertility and sterility in this study. Here, we dissect the phenotypes presented in *amx-1* mutants to investigate how AMX-1 contributes to fertility defects. AMX-1 is necessary for normal fertility and the absence of its expression exhibits a decreased brood size. However, in contrast to mammalian LSD1 homolog SPR-5, AMX-1 is not essential for transgenerational fertility defects. In addition, *amx-1* mutants exhibit mild but significant sterility. *amx-1* mutants retained a reduced number of sperms and produced sterile worms that lack germline or sperms. Interestingly while p53/CEP-1 is dispensable for brood size reduction, the sterility in *amx-1* mutants requires p53/CEP-1 function. Also, the lack of gametes exhibited in *amx-1* mutants correlates with upregulation of Piwi expression and desilencing of a subset of transposons. Altogether, our study demonstrates that lack of AMX-1 induced sterility in a p53/CEP-1 dependent manner.

## Materials and Methods

### Strains and Alleles

*C. elegans* strains were cultured at 20°C under standard conditions as described in Brenner (Brenner, 1974). The N2 Bristol strain was used as the wild-type background. The following mutations and chromosome rearrangements were used in this study: LGI: *spr-5(by101), cep-1(lg12501)*, *prg-1(n4357),* hT2[*bli-4(e937) let-?(q782) qIs48*](I; III); LGIII: *amx-1 (ok659), qC1[dpy-19(e1259) glp-1(q339) qIs26*] (III). The *amx-1* deletion mutant (*ok659*) was provided by the *C. elegans* Gene Knockout Project at the Oklahoma Medical Research Foundation, which was part of the International *C. elegans* Gene Knockout Consortium. *amx-1* mutant *(ok659)* carries a 2636 bp deletion encompassing the entire SWIRM domain and most of the amine oxidase domain (Zhang et al., 2021). *amx-1(ok659)* worms were outcrossed to the wild-type N2 and balanced with qC1 with roller and GFP markers for maintenance.

### Quantitative Analysis

Statistical comparisons between genotypes were performed using the two-tailed Mann-Whitney test, 95% confidence interval (C.I.) unless otherwise specified.

### DNA Damage Sensitivity

Young adult homozygous *amx-1* animals were picked from the progeny of *amx-1*/qC1 parent animals. For HN2 sensitivity, 24 h post L4 hermaphrodites were treated with 0, 150 μM of HN2 in M9 buffer containing *E. coli OP50* with sharking at 50 rpm in dark for ∼20 h. After the DNA damage treatments, animals were washed with M9 containing Triton X100 (100 ml/L) (Kim and Colaiácovo, 2014; Kim and Colaiácovo, 2015a).

Hatching sensitivity (embryonic lethality) was examined in >24 animals 3–4 h after drug treatment. For all other damage sensitivity experiments, >24 animals were plated, 7 per plate, and hatching was assessed for the period of 20–24 h following treatment. Each assay was replicated at least twice in an independent experiment.

### Sterility and Fertility

Young adult homozygous *amx-1* animals were picked from the progeny of *amx-1*/qC1 parent animals. To monitor fertility and sterility, brood size was scored corresponding to each worm’s total number of eggs. For fertility analysis, at least four hundred worms were analyzed for each genotype at each generation reported. Worms produced ∼100 or fewer progenies were counted as small brood size (SBS). For sterility analysis, we scored sterility when worms produced no viable progenies until two days after L4 stage. At least a thousand worms were analyzed for each genotype at each generation reported in three independent biological replicates.

HN2 sensitivity was assessed by placing age matched L4 hermaphrodites on seeded NGM plates containing 0, 50, or 100 μM of HN2 (mechlorethamine hydrochloride). We scored fertility until 2 days after L4 stage. HU sensitivity was assessed by placing animals on seeded NGM plates containing either 0 or 2 mM HU. We scored fertility until two days after L4 stage (Kim and Colaiácovo, 2015b).

### Carnoy’s Fixation

We prepared hermaphrodite worms with Carnoy’s solution instead of dissecting them to avoid potential artifacts and preserve internal organs and tissues. In brief, worms were fixed on positively charged slides with Carnoy’s solution (6 ml ethanol, 3 ml acetic acid and 1 ml chloroform). Slides were air-dried at least 2 h and stained with ∼2 μg/ml of DAPI for >20 min before analysis.

### Immunofluorescence Staining

Whole-mount preparations of dissected gonads, fixation and immunostaining procedures were conducted as described in (Batista et al., 2008). Primary antibody was used at the following dilutions: mouse anti-H3K4me2 (1:250, Millipore CMA-303). Secondary antibody used was: Cy3 anti-mouse (1:250) from Jackson lmmunoresearch.

Immunofluorescence images were collected at 0.2 μm intervals with a Nikon Eclipse Ti2-E inverted microscope and a DSQi2 camera. Photos were taken with a 60 × objective in combination with or without 1.5 × auxiliary magnification. Partial projections of half-nuclei were shown. Images were subjected to deconvolution by using the NIS Elements software (Nikon).

To examine AMX-1 histone demethylase level *in vivo*, dissected gonads from *amx-1* age-matched adult hermaphrodites were probed for H3K4me2. To ensure that both control and mutant animals were processed and imaged under identical conditions, we dissected the both types of animals on the same slide as described in Zhang et al. (2021). Fluorescent intensity signal is sampled from multiple nuclei from different gonads and quantitated by using ImageJ or GelQuantNet.

### Quantitative Real-Time PCR

cDNA was produced from young hermaphrodite worm RNA extracts using the ABscript II First synthesis (ABclonal RK20400). Real-time quantitative PCR amplification for were carried out using ABclonal 2X SYBR Green Fast mix (Abclonal RK21200). Amplification was conducted in a LineGene 4800 (FQD48A BIOER) with initial polymerase activation at 95˚C for 2 min, followed by 40 cycles of: 95˚C for 15 s denaturation, 60˚C for 20 s for annealing and elongation. After 40 cycles, a melting curve analysis was carried out (60 to 95˚C) to verify the specificity of amplicons. Tubulin encoding *tba-1* gene was selected for a reference gene based on *C. elegans* microarray expression data (Hoogewijs et al., 2008). Each qPCR has been repeated independently at least three times. Primer sequences are listed in Supplemental Table S1.

## Results

### AMX-1 Is Required for Proper Fertility but Dispensable for Transgenerational Fertility Defects

**FIGURE 1:**
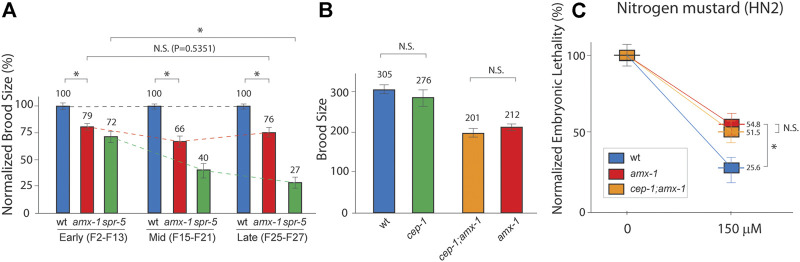
AMX-1 is necessary for proper fertility but not for transgenerational fertility defects. **(A)** Brood size is scored among the progenies of indicated genotypes. *amx-1* mutants exhibit the reduced brood size compared to wild type in early [F2-F13, *p* < 0.0001), mid (F15-F21), *p* < 0.0001) or late generations(F25-27), *p* < 0.0001]. However, no obvious transgenerational decline was observed between early and late generations of *amx-1* mutants (dashed lines, *p* = 0.5351 in early vs. late generations of *amx-1* and *p* = 0.0022 in early vs. late generations of *spr-5*. *p* = 0.1807 for wt and *cep-1, p* = 0.2770 for *amx-1* and *cep-1;amx-1*. **(B)** Brood size comparison between indicated genotypes. **(C)** Relative hatching of wild type, *amx-1* and *cep-1;amx-1* mutants after treatment with the indicated dose of nitrogen mustard (HN2). Asterisk indicates statistical significance. Error bars represent standard errors of the mean. n > 24 for each genotype. N.S. indicates no statistical significance. *p* values calculated by the two-tailed Man-Whitney test, 95% C.I.

Previously, both histone demethylases AMX-1 and SPR-5 exhibited brood size reduction (Katz et al., 2009; Zhang et al., 2021). Consistent with these reports, *amx-1* and *spr-5* null mutants exhibited a 21% and 28% reduction compared with the wild type, respectively (Figure 1A, 100% vs. 79% and 100% vs. 72% in early generations). Additionally, *spr-5* exhibited a progressive fertility defect in which brood size decreases over generations [green dashed line; 72%, 40%, 27% in early, mid, late generations, respectively, *p* = 0.0022 in early and late generations of *spr-5* (Katz et al., 2009)]. On the contrary, *amx-1* did not alter fertility in either mid or late generations compared to early generations, suggesting that AMX-1 is dispensable for transgenerational fertility defects (Figure 1A, red dashed line; 79, 66, 76 in early, mid, late generations, *p* = 0.5351 in early and late generations of *amx-1*). Given that *amx-1* is required for ICL (intercrosslink) repair (Zhang et al., 2021), we further analyze how AMX-1 contributes to defective fertility.

### p53/CEP-1 Is Not Required for Fertility Defects Exhibited in *amx-1* Mutants

Lack of AMX-1 expression leads to higher-level p53/CEP-1-dependent DNA damage apoptosis [Supplemental Figure S1, (Zhang et al., 2021)]. Thus, we further assessed whether the reduced brood size observed in *amx-1* requires a p53/CEP-1-mediated DNA damage pathway. We found no distinct difference between wild-type and *cep-1* mutants (Figure 1B, 305 vs. 276, *p* = 0.1807 by Mann-Whitney test). Similarly, *cep-1;amx-1* double mutants did not alter brood size significantly, indicating that reduced fertility in *amx-1* does not require the p53/CEP-1 pathway (201 vs. 210 in *cep-1;amx-1* and *amx-1*, *p* = 0.2770).

Previously, *amx-1* has been implicated in the activation of DNA damage and is required for proper ICL repair by modulating mismatch repair gene expression (Zhang et al., 2021). Thus, next, we examined whether p53/CEP-1 is necessary for ICL sensitivity. Similarly, we found that lack of p53/CEP-1 did not alter the embryonic-lethality level upon nitrogen mustard exposure, further supporting that CEP-1 is dispensable for fertility defects displayed in *amx-1* mutants (Figure 1C, 54.8 vs. 51.5 in *amx-1* and *cep-1;amx-1, p* = 0.3001; 54.8 vs. 25.6 in *amx-1*, and wild type*, p* = 0.0036). Together, these observations suggest that the lack of AMX-1 expression results in non-progressive fertility defects in a p53/CEP-1-independent manner.

### Lack of AMX-1 Exhibits Non-Transgenerational Sterility and SBS Phenotype

**FIGURE 2:**
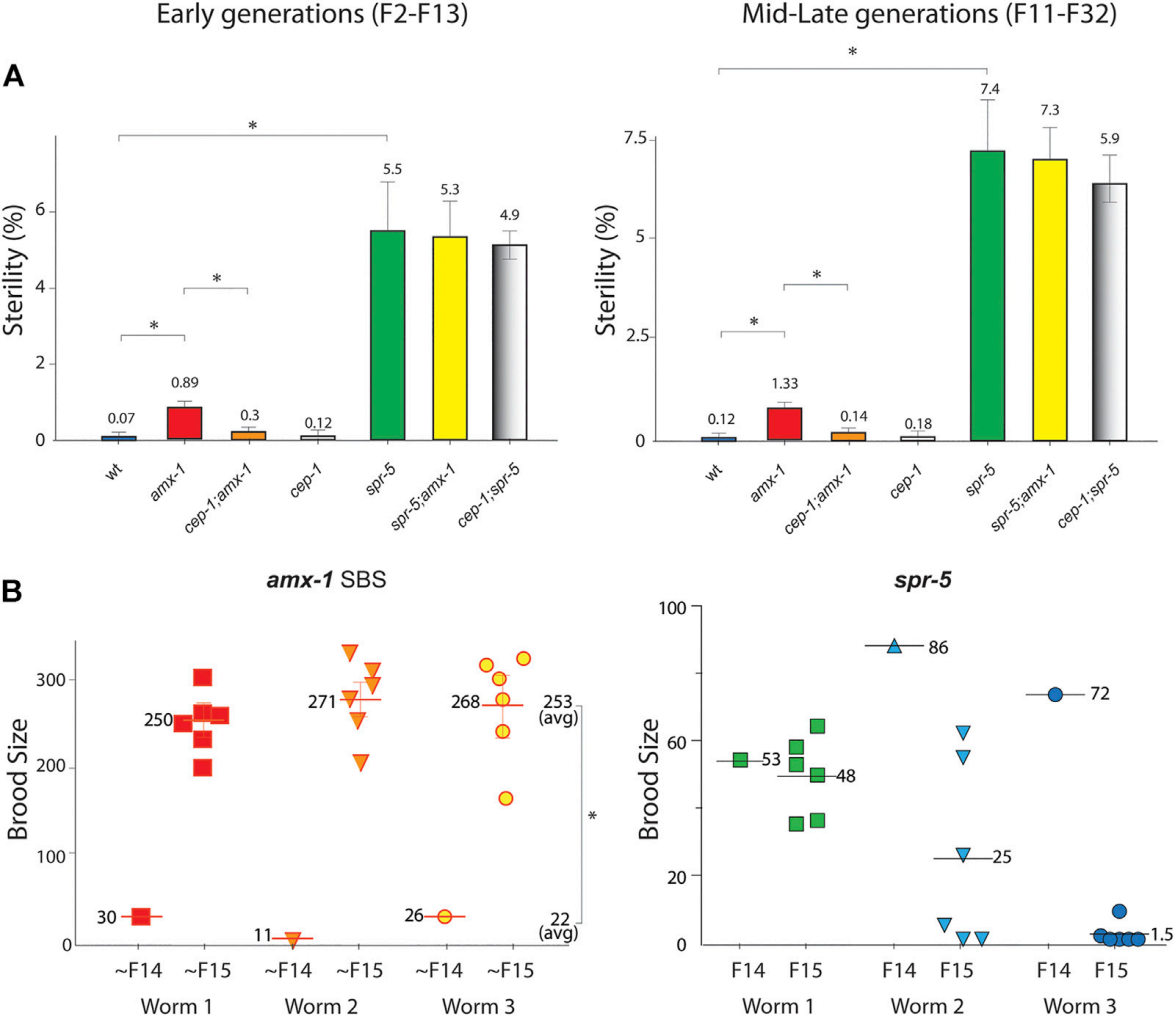
Lack of *amx-1* expression leads to non-transgenerational sterility in a CEP-1-dependent manner. **(A)** Sterility is scored among the progenies of indicated genotypes. *amx-1* mutants exhibit sterility (zero brood size) compared to wild type in early (F2-F13) or mid-late generations (F11-F32). No obvious transgenerational decline was observed between early and mid-late generations of *amx-1* mutants (*p* = 0.0006 in early vs. mid-late generations of *amx-1* and *p* = 0.0036 in early vs. late generations of *spr-5*). Asterisks indicate statistically significant changes. Error bars represent standard errors of the mean. n > 24 for each genotype. At least a thousand worms are analyzed for each genotype at each generation reported in three independent biological replicates. **(B)** Brood size is scored among the progenies of three independent SBS (small brood size) *amx-1* mutants. average brood size 22 vs. 253 in ∼F14 and ∼F15 of *amx-1*, *p* = 0.0097; 70 vs. 25 in F14 and F15 of *spr-5*, respectively, *p* = 0.0233, Mann-Whitney test.

In addition to reduced fertility, lack of SPR-5 expression exhibited a sterility phenotype that produced no viable progeny (Katz et al., 2009). Consistent with the report, lack of SPR-5 led to sterility which became more severe with generations (Figure 2A, 5.5 vs. 7.4% in early and mid-late generations, *p* = 0.0006 and *p* = 0.0036 in early and mid-late generations of wild type and *spr-5,* respectively).

Interestingly, *amx-1* mutants displayed significantly higher sterility than the wild type. Specifically, early generations of *amx-1* exhibited 12.7-fold higher sterility than the wild type (Figure 2A, 0.07 vs. 0.89%, *p* = 0.0104 in early generations). Similarly, mid-late generations of *amx-1* showed an 11-fold induction compared to the wild type, supporting the idea that the sterility in *amx-1* mutants is not transgenerational (0.12% vs. 1.33%, *p* = 0.0251 in wt and *amx-1;* 0.89% vs. 1.33%, *p* = 0.5657 in early vs. mid-late generations of *amx-1*).

In addition to sterility, *amx-1* mutants often produce ∼100 or fewer progenies, hence the name ‘small brood size’ (SBS, ∼6%). Interestingly, the SBS phenotype of *amx-1* mutants does not maintain the reduced fertility over generations; it resets in their following generation, further confirming that *amx-1*’s sterility is not likely imprinted for the subsequent progenies (Figure 2B, average brood size 22 vs. 253 in ∼F14 and ∼F15, respectively, *p* = 0.0097, Mann-Whitney test). In contrast, *spr-5* mutants displayed a progressive brood size reduction, as reported previously [average brood size 70 vs. 25 in F14 and F15, respectively, *p* = 0.0233, (Katz et al., 2009)].

### Lack of AMX-1 Leads to p53/CEP-1 Dependent Sterility

Since AMX-1 functions in ICL damage repair (Zhang et al., 2021), we further tested whether a key regulator of DNA damage-induced checkpoint p53/CEP-1 is essential for sterility observed in the *amx-1* mutants.

*cep-1* mutants exhibited no obvious sterility compared to wild-type control regardless of early, mid, or late generations suggesting that CEP-1 is dispensable for sterility (Figures 2A, 0.07% vs. 0.12%, *p* = 0.8708 in early generations; 0.12 vs. 0.18%, *p* > 0.9999 in mid-late generations between wild type and *cep-1*). Interestingly, lack of p53/CEP-1 expression in *cep-1;amx-1* double mutants suppresses sterility observed in the *amx-1* mutants in both early and mid-late generations, indicating that p53/CEP-1 is required for sterility exhibited in *amx-1* mutants (Figure 2A, 0.89 vs. 0.3%, *p* = 0.0026 in early generations; 1.33% vs. 0.14%, *p* = 0.0423 in mid-late generations).

We further examined whether *cep-1* suppresses sterility in *spr-5* mutants. However, unlike *amx-1*, sterility displayed in *spr-5* was not decreased in the *cep-1;spr-5* double mutants suggesting that p53/CEP-1 is dispensable in *spr-5* (Figure 2A, 5.5 vs. 4.9, *p* > 0.9999 in early generation; 7.4 vs. 5.9, *p* = 0.9360 in mid-late generations). Also, in line with this idea, *spr-5;amx-1* double mutants were not considerably altered compared to *spr-5* single mutants implying that sterility phenotypes of two histone demethylases may stem from independent pathways (5.5 vs. 5.3, *p* = 0.9383 in early generations; 7.4 vs. 7.3, *p* = 0.3359 in late generations). Taken together, these observations suggest that the lack of AMX-1 induces p53/CEP-1 dependent sterility.

### Sterile Worms Lack Germline Nuclei and Embryos.

**FIGURE 3:**
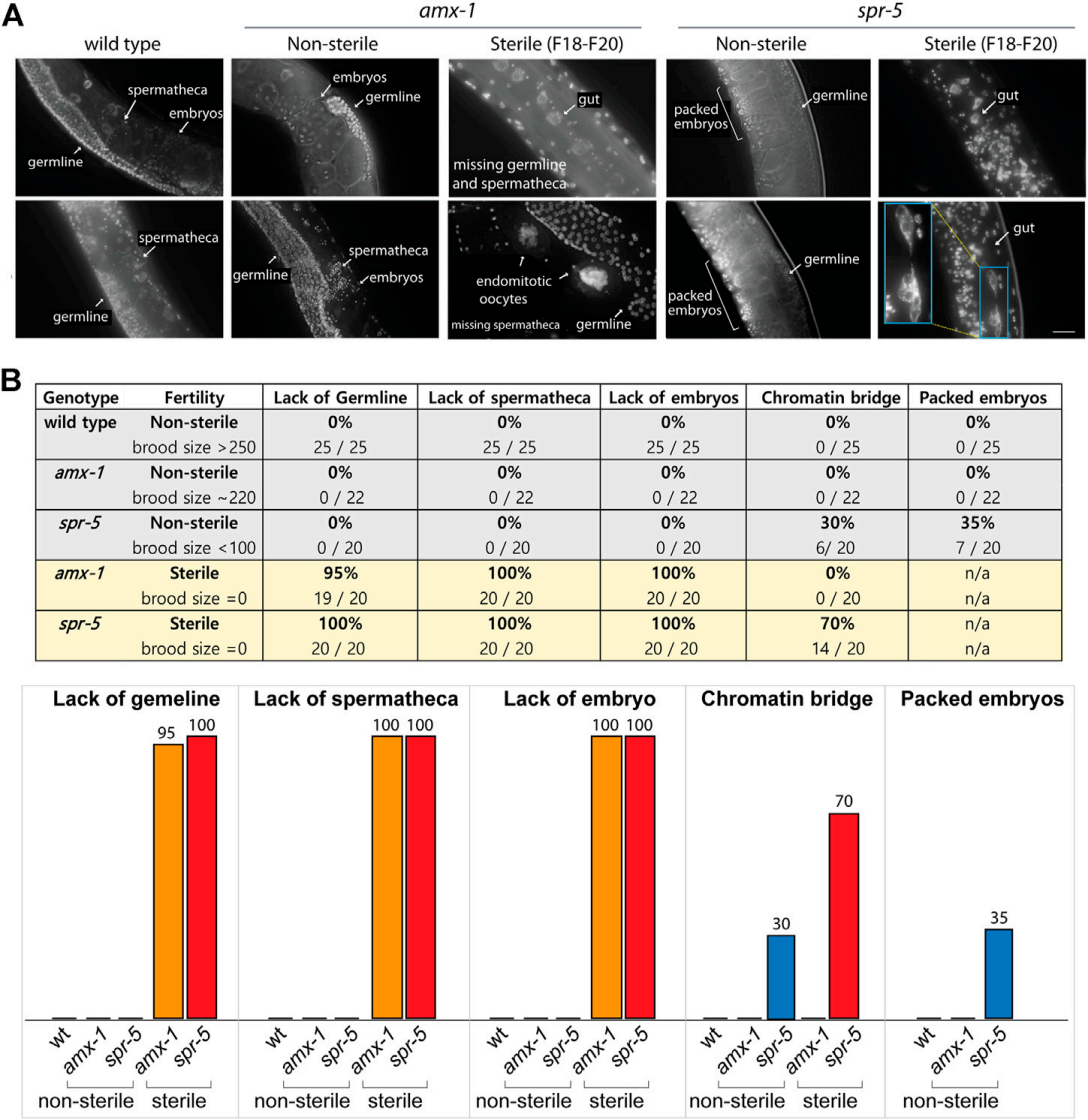
Lack of AMX-1 leads to sterility with defective germline development. **(A)** Representative images of the worms exhibiting sterility. Wild type N2, non-sterile *amx-1* and *spr-5* are shown as control. While wild type and non-sterile *amx-1* shows embryos, spermatheca and germline, sterile *amx-1* mutants fail to exhibit germline nuclei or spermatheca, indicating the defective gamete development. Bar, 10 μm. **(B)** Quantification of the phenotypic defects observed in whole worms without dissection. Inset (blue) shows an example of chromatin bridge of gut cells. Arrows indicate internal compartments of hermaphrodites. N > 20 worms for all genotypes.

To further investigate and analyze the phenotypes of worms displaying sterility, we performed a comparative morphological analysis using fluorescent staining. We utilized whole worms without dissection to avoid potential artifacts while preparing worms for slides. As expected, wild type N2 worms exhibit germline nuclei, embryos and spermatheca (Figures 3A,B). Similarly, non-sterile *amx-1* mutants contain embryos, germline nuclei and spermatheca. In contrast, interestingly, sterile *amx-1* mutants exhibit an absence of germline and/or sperms, suggesting that the sterility likely stems from the defective gamete development (Figures 3A,B, 95% and 100%, respectively). Furthermore, sterile worms lack fertilized embryos (100%, 20 out of 20 worms) and exhibit unfertilized endomitotic embryos (Figure 3A, 5%, 1 out of 20) further supporting the idea of defective gamete formation in *amx-1* mutants.

Non-sterile *spr-5* mutants, which display a 28% reduction in brood size (Figure 1A), contain germline nuclei, spermatheca, and fertilized eggs. However, we frequently found their embryos packed tightly inside the body (35%, 7 out of 20) in contrast to *amx-1* or wild type worms. While non-sterile *spr-5* mutants contain both germline and sperms/spermatheca, sterile *spr-5* mutants lack both sperms and oocytes (100%, 20 out of 20). In addition, they frequently exhibited chromatin bridges (70%, 14 out of 20), which were not presented in the *amx-1* mutants. Our observations suggest that defective germ cell development leads to sterility in *amx-1*.

### Rescue of *Amx-1* Sterility by Crossing With Wild Type

**FIGURE 4:**
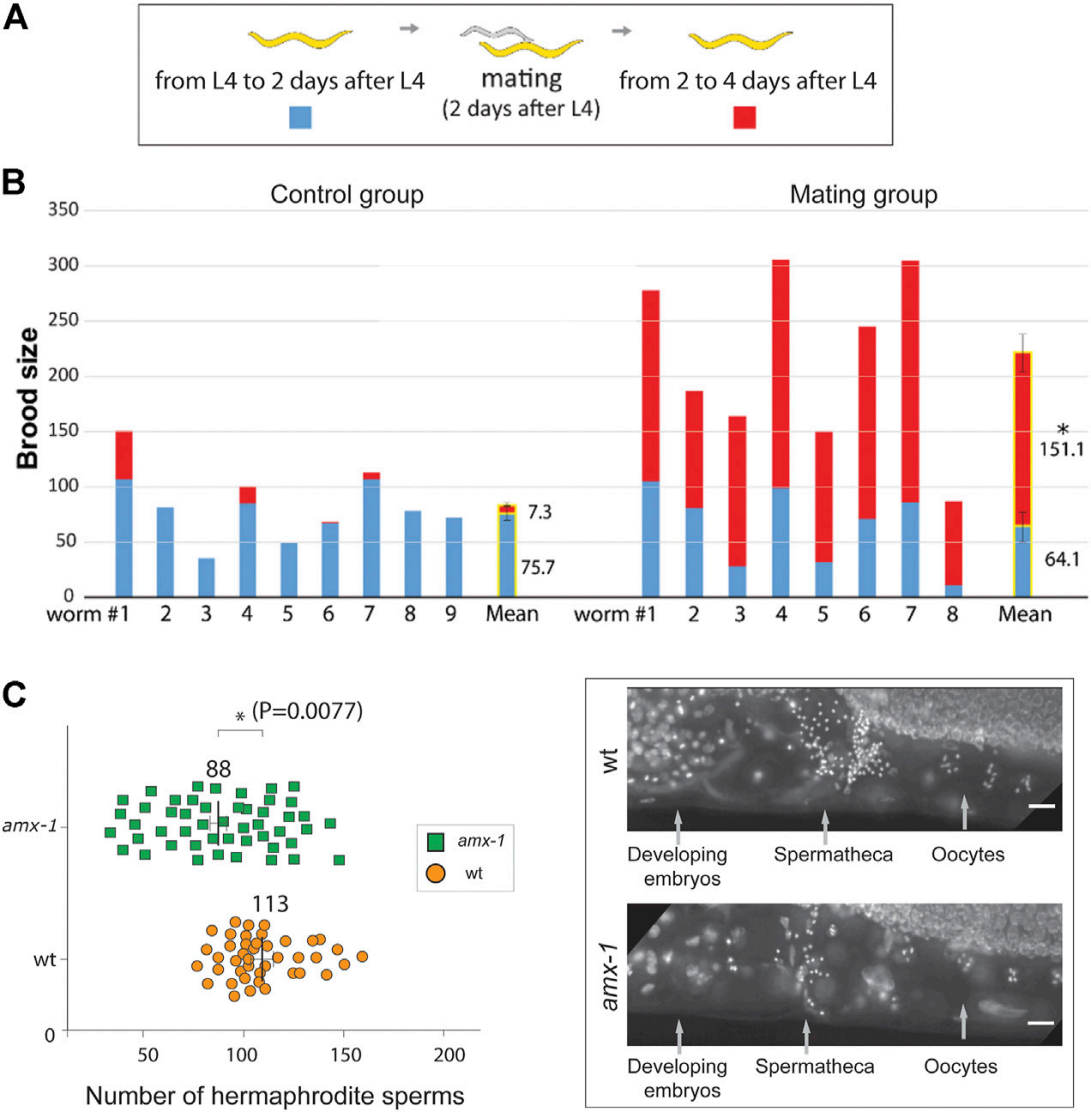
Mating rescues the SBS phenotype of *amx-1*, retaining fewer sperms. **(A)** Schematic representation of assay for determining the number of progenies before and after mating. We counted the number of progenies from the L4 stage worms for 4 days. Starting from L4 stages, we counted the number of progenies for 2 days. Thereafter, worms were mated with wild type and we continued to monitor the number of progenies for the following two more days. **(B)** Quantification of the number of brood sizes before and after mating. Individual worms and mean values of brood size with or without mating are shown. *n* = 9 for control and *n* = 8 for mating. Error bars represent standard errors of the mean. **(C)** Left, quantification of the number of hermaphrodite sperms per gonad arm. Right, representative images of the worms containing spermathecae. Raw data is available in Supplementary Figure S1. Error bars represent standard errors of the mean. *n* = 43 for wild type and *n* = 48 for *amx-1*. Asterisks indicate statistically significant changes by the two-tailed Man-Whitney test, 95% C.I. Bars, 10 μm.

Our observations suggest that the absence of germ cells leads to the sterility of *amx-1.* To further examine the importance of germ cell contribution *in vivo*, we crossed SBS *amx-1* mutants with wild type and obtained progeny heterozygous for *amx-1* (Figure 4A). Before mating, no distinct difference was observed in the control or the mating groups (Figure 4B, 75.7 vs. 64.1 from L4 to 2 days after L4, *p* = 0.4391). However, mating increased the brood size by twentyfold, indicating that fertility defects originate from defective sperm development (7.3 vs. 151 from two to 4 days after L4, *p* < 0.0001, Mann-Whitney test).

In line with this observation, we found a significant reduction in the number of sperm of *amx-1* mutants compared to the control (Figure 4C and Supplementary Figure S2, 88 vs. 113 in *amx-1* and wild type respectively, *p* = 0.0077). Our genetic and cytological analysis demonstrated that defective sperm development contributes to the sterility of *amx-1* mutants.

### p53/CEP-1 Dependent Sterility

p53/CEP-1 is a transcription factor of DNA damage-induced apoptosis and its signaling has been implicated in multiple pathways, including cell cycle checkpoint, senescent, angiogenesis and differentiation (Hafner et al., 2019). *C. elegans* has only one p53 gene CEP-1 (Derry et al., 2001; Schumacher et al., 2001); thus the lack of CEP-1 leads to failure in DNA damage pathways (Kim and Colaiácovo, 2014; Kim and Colaiácovo, 2015a).

**FIGURE 5:**
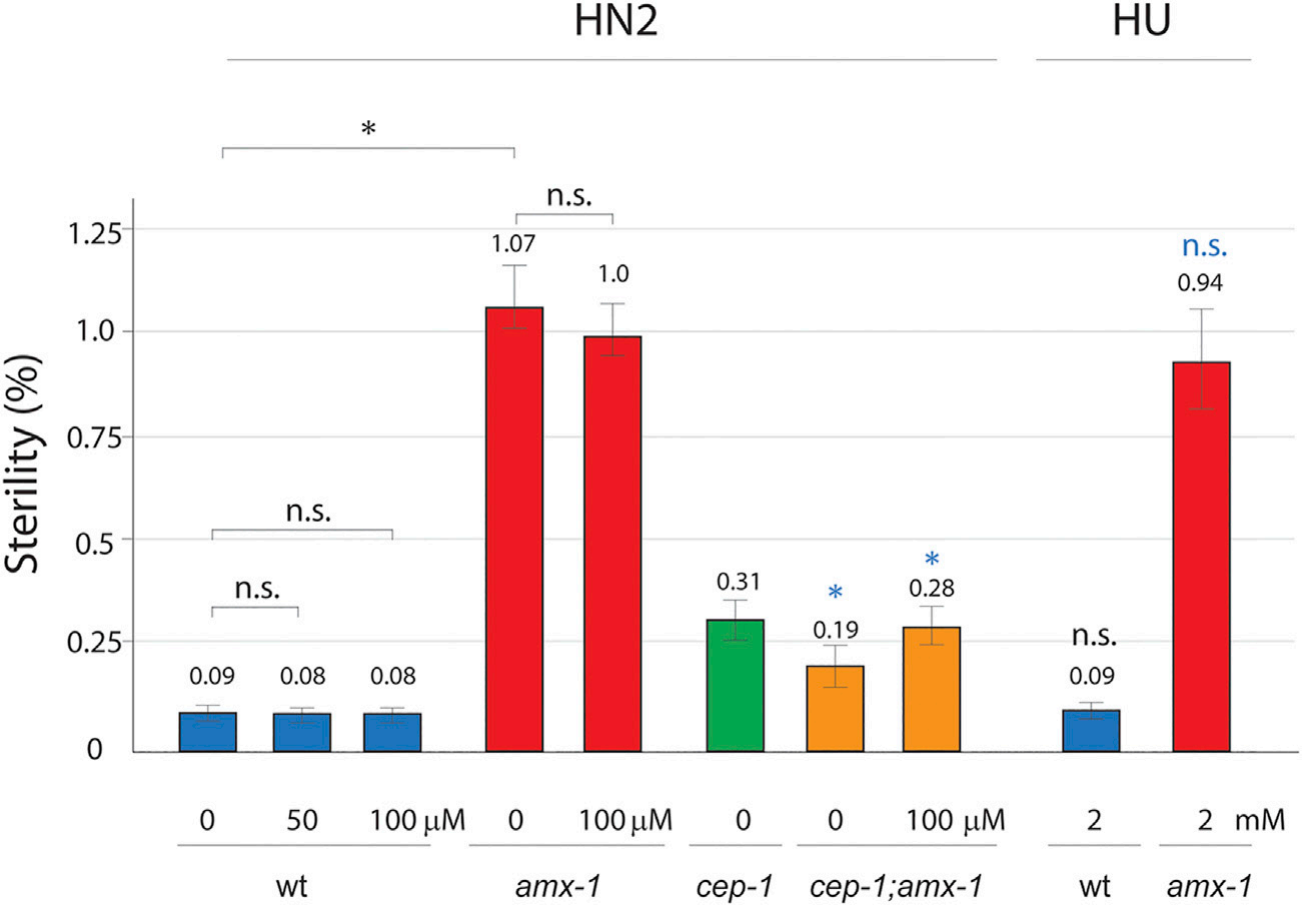
Sterility requires DNA damage-independent function of p53. Sterility was scored among the progenies of indicated genotypes with or without DNA damage treatment. Worms were incubated with or without HN2 (nitrogen mustard) and HU (hydroxy urea) treatment at indicated doses (0, 50, 100 μM HN2, and 0, 2 mM HU). Asterisks indicate statistically significant changes. Black colored asterisks or n.s indicate statistic comparison over wild type. Blue colored asterisks or n.s. indicate statistic comparison over *amx-1* mutants. Error bars represent standard errors of the mean. At least three independent biological replicates have been performed. *n*= 1000–3624 worms for each analysis.

Given that p53/CEP-1 engaged in multiple pathways and the lack of p53/CEP-1 suppresses the sterility observed in *amx-1* mutants (Figure 2A), we further evaluated whether the DNA damage pathway of p53/CEP-1 contributes to the sterility. For this purpose, we exposed young adult hermaphrodites to media containing DNA damaging agents and monitored the embryonic lethality as described previously (Kim and Colaiácovo, 2015a; Kim and Colaiácovo, 2015b). Consistent with the data presented in Figure 2A, *amx-1* mutants exhibited ∼10-fold higher sterility compared to wild type control (Figure 5, 1.07 vs. 0.09% respectively, *p* = 0.0034).

Interestingly, wild type exposed in 50 μM nitrogen mustard (HN2), which produces interstrand crosslinks (ICLs), did not induce sensitivity compared to control (Figure 5, 0.09 vs. 0.08 in wt and wt + HN2, *p* = 0.5000) suggesting that ICL DNA damages may not affect sterility. Similarly, at a higher dose of HN2, where wild type worms displayed 24% embryonic lethality (Zhang et al., 2021), both wild type and *amx-1* did not alter the level of sterility (0.09 vs. 0.08 in wt and wt + HN2, *p* = 0.5000; 1.07 vs. 1.0 in *amx-1* and *amx-1* + HN2, *p* = 0.1558 at 100 μM), indicating that ICL damages did not initiate sterility in *amx-1* mutants. Furthermore, hydroxyurea (HU), which induces DNA replication stress by depleting dNTP pools, did not alter sterility in either wild type or *amx-1* mutants, further supporting the idea that DNA damage repair is dispensable for sterility exhibited in *amx-1* (0.09 vs. 0.09 in wt and wt + HU, *p* = 0.4354; 1.07 vs. 0.94 in *amx-1,* and *amx-1*+HU, *p* = 0.1966).

Next, we examined whether p53/CEP-1 is required for sterility upon ICL damages. While *cep-1* single mutant did not represent distinct changes over wild type (0.31 vs. 0.09 in *cep-1* and wt, *p* = 0.2086), *cep-1;amx-1* double mutants suppressed sterility of *amx-1* either in the presence or absence of HN2 treatment, suggesting that CEP-1 is required for the sterility of *amx-1* regardless of ICL DNA damage (82% reduction, 1.07 vs. 0.19 in *amx-1* and *cep-1;amx-1*, *p* = 0.0034; 72% reduction, 1.0 vs. 0.28 in *amx-1*+HN2 and *cep-1;amx-1* + HN2, *p* = 0.0442). Altogether, these observations suggest that ICL damage is dispensable for the sterility of *amx-1*, but the sterility requires function of CEP-1.

### p53/CEP-1 Mediated Upregulation of Piwi Expression

Since the sterility of *amx-1* mediated p53/CEP-1 function, we further examined the possible cause of sterility*.* Piwi is essential for fertility and is implicated in transposable elements silencing. Piwi genes function in germline stem cells, stem cell development, gametogenesis and RNA interferences in diverse organisms. *C. elegans*, Piwi/*prg-1* mutants consistently exhibited various germline abnormalities including empty germlines (Das et al., 2008; Heestand et al., 2018). The absence of germline phenotype presented in *amx-1* sterile mutants reminds us of Piwi, which are essential for the normal germline development of various species. *C. elegans* Piwi/PRGs are required for transposon silencing and the null mutants induced upregulation of transposons as well as transpositions (Das et al., 2008).

**FIGURE 6:**
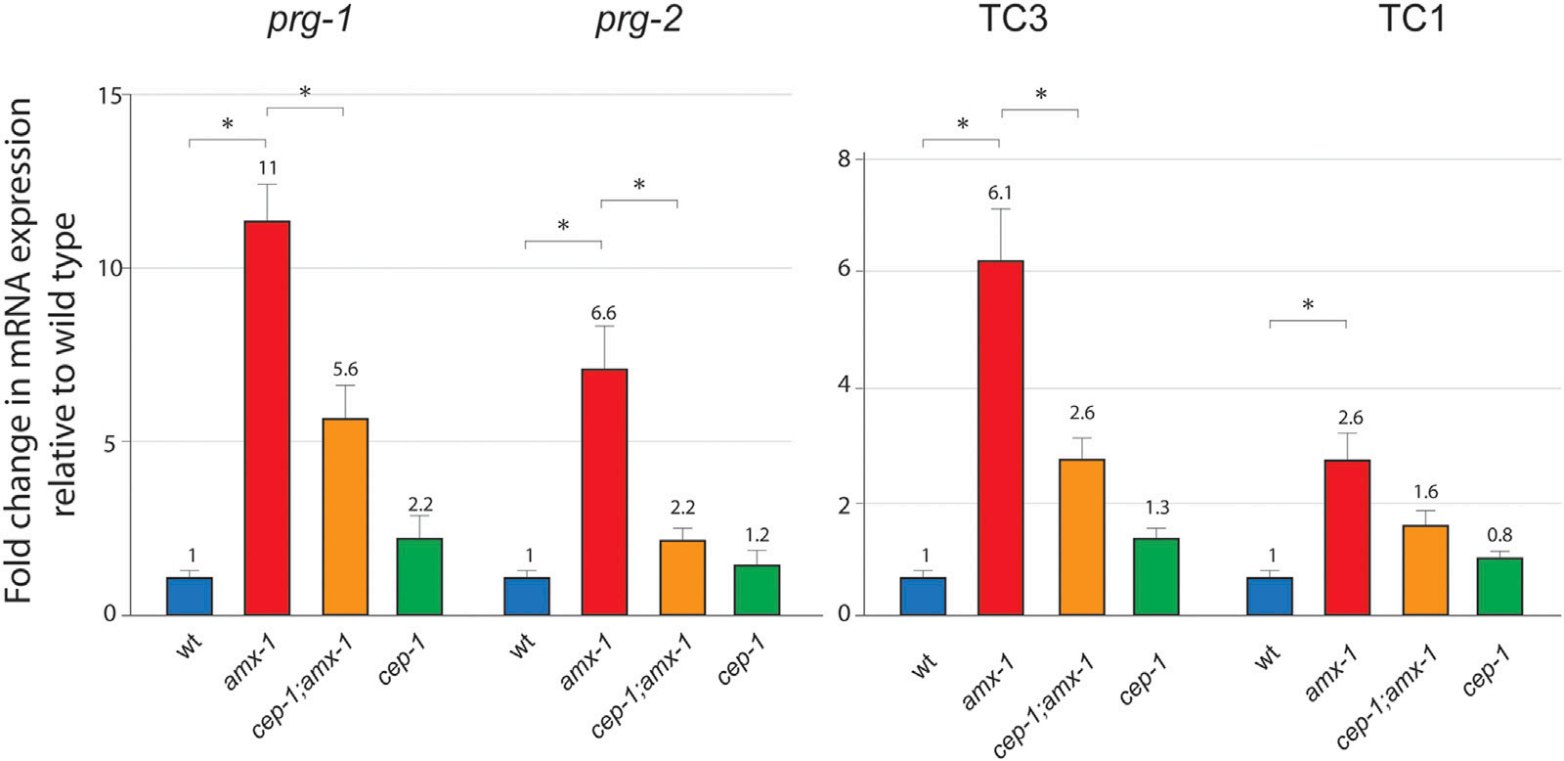
AMX-1 is required for the proper expression of Piwi and transposon silencing. Changes of relative mRNA expression levels of Piwis (*prg-1* and *prg-2*) and transposons (Tc3 and Tc1). Lack of *amx-1* expression increases Piwi expression. mRNA expression was monitored with qPCR primers spanning exon-exon junctions for each indicated genes. The data shown are the means with SEM analyzed employing an unpaired, two-tailed *t*-test. Assay repeated at least three independent sample preparations. Tubulin encoding *tba-1* was used as an endogenous reference. Asterisks indicate significant changes of mRNA expression.

Surprisingly, the mRNA expression level of *prg-1* or *prg-2* was significantly higher in *amx-1* mutants than in wild-type (Figures 1, 6, 1 vs. 11 in *prg-1* expression, *p* = 0.0022; 1 vs. 6.6 in *prg-2* expression, *p* = 0.0033). The induced level of *prg-1* or *prg-2* was significantly suppressed in *cep-1;amx-1* double mutants, suggesting the p53/CEP-1 mediated Piwi expression in *amx-1* mutants (11 vs. 5.6 of *prg-1* expression, *p* = 0.0095; 6.6 vs. 2.2 of *prg-2* expression, *p* = 0.0049). Mild elevations found in *cep-1* single mutants did not significantly alter mRNA expression for *prg-1* and *prg-2* (*p* = 0.1199 and *p* = 0.6396, respectively). Also, no obvious changes were observed between *cep-1* and *cep-1;amx-1* double mutants in both transposons (2.6 vs. 1.3, *p* = 0.1198 in Tc3; 1.6 vs. 0.8, *p* = 0.1482 in Tc1). Altogether, our mRNA analysis demonstrated that AMX-1 is required for the proper expression of Piwi genes in a CEP-1 dependent manner.

PRG-1 and PRG-2 are required for transposon silencing in *Drosophila* and *C. elegans* (Aravin et al., 2001; Vagin et al., 2006; Das et al., 2008). To examine transposon silencing in *amx-1* mutants, we analyze two major DNA transposons, Tc1 and Tc3, using primers specific for fifteen Tc1 and 20 Tc3 loci (Das et al., 2008). Despite the higher level of expression of *prg-1* and *prg-2*, both Tc3 and Tc1 were significantly upregulated in *amx-1* mutants compared to the level of wild types, suggesting that the lack of AMX-1 leads to the sterility mediated by transposon desilencing (Figures 1, 6, 1 vs. 6.1 in Tc3 expression, *p* = 0.0002; 1 vs. 2.6 in Tc1 expression, *p* = 0.0358). Also, consistent with Piwi expression data, *cep-1;amx-1* double mutants suppressed the increased Tc3 level presented in *amx-1* significantly, indicating that CEP-1 is required for Tc3 desilencing (2.6 vs. 6.1, *p* = 0.0078). However, no distinct suppression was observed in Tc1 expression, indicating that CEP-1 is specific to Tc3 desilencing (1.6 vs. 2.6, *p* = 0.0689).

### The Level of H3K4me2 Was Not Altered in Sterile or SBS Worms

Given that *amx-1* mutants showed a higher level of H3K4me2 in embryos and germline nuclei compared to wild types, and higher H3K4 methylation levels in *spr-5* mutants contribute to transgenerational fertility, we further explored whether the sterility of *amx-1* mutants is the outcome of the extra accumulation of dimethylation (Katz et al., 2009; Greer et al., 2014; Zhang et al., 2021).

For this purpose, dissected gonads from *amx-1* mutants exhibiting non-sterile (> 200 brood size) or sterile age-matched adult hermaphrodites were probed for H3K4me2. Consistent with previous reports, *amx-1* is required for the proper level of H3K4me2 compared to wild type N2 [(Wang et al., 2020; Zhang et al., 2021), Figure 7A, B, 71 vs. 100 in PMT, *p* = 0.0072; 67 vs. 100 in pachytene, *p* = 0.0032; 76 vs. 100 in embryos, *p* = 0.0075]. However, sterile *amx-1* mutants did not alter the H3K4me2 signal compared to the non-sterile worms, suggesting that sterility in *amx-1* was not the consequence of H3K4me2 accumulation in the germline nuclei (100 vs. 110 in PMT, *p* = 0.5732; 100 vs. 98 in pachytene, *p* = 0.9931). Lastly, since sterile *amx-1* animals lack fertilized eggs, as shown in Figure 3, we employed SBS embryos to measure the histone methylation level. No distinct difference in the methylation level between SBS and the non-sterile group was detected (100 vs. 101 in embryos, *p* = 0.8456).

**FIGURE 7:**
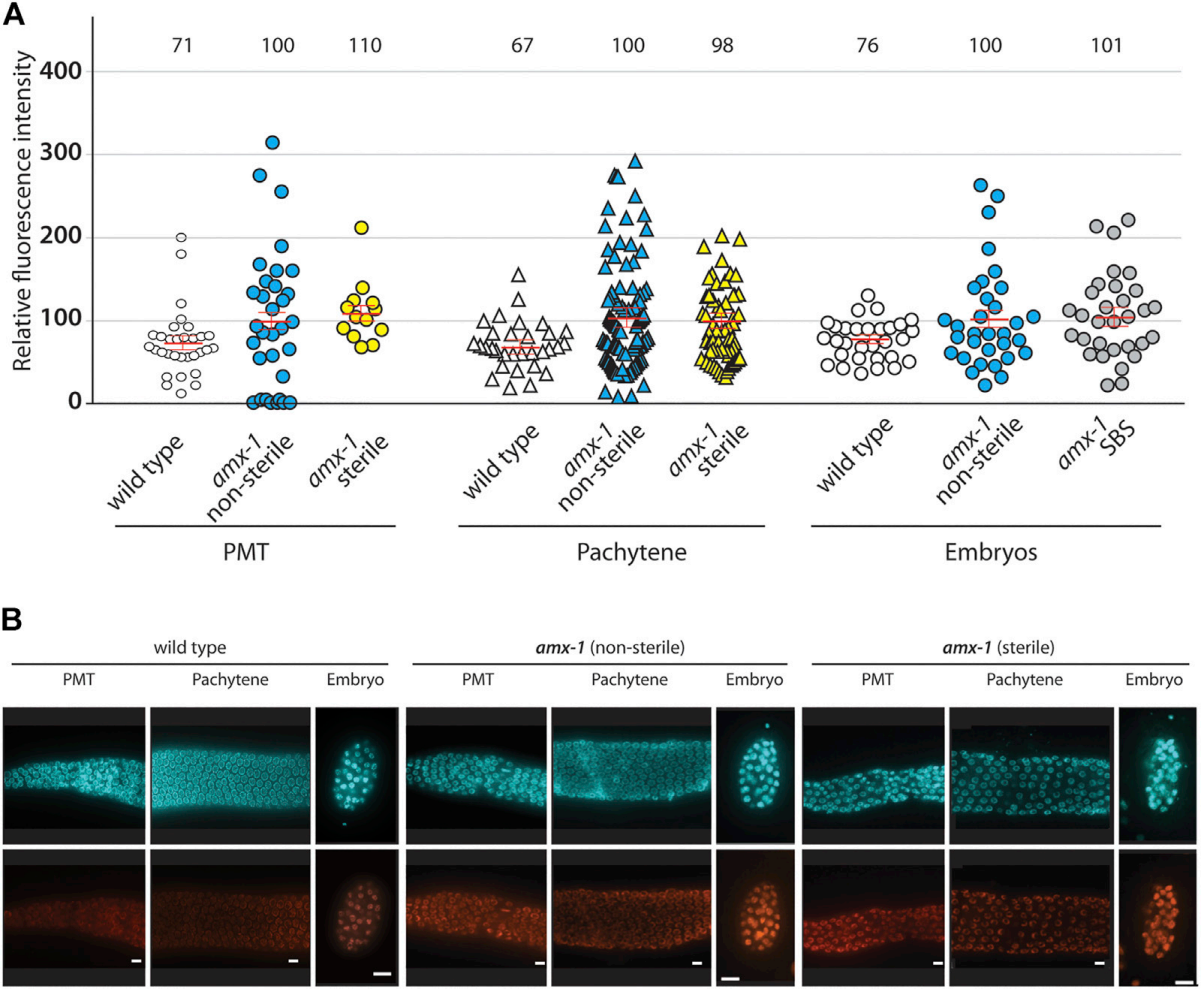
Sterile worms do not alter the level of H3K4me2 in both mitotic and meiotic germline. **(A)** While *amx-1* mutants altered H3K4me2 level compared to wild type (in PMT, *p* = 0.0072; in pachytene, *p* = 0.0032; in embryo, *p* = 0.0075), no significant change was detected in nuclei between non-sterile and sterile worms (in PMT, *p* = 0.5732; in pachytene, *p* = 0.9931; in embryo, *p* = 0.8456). Individual nuclei were sampled for fluorescence intensity using ImageJ. The bars represent the mean fluorescence intensity. N = 5–6 worms. **(B)** Representative images used for the analysis in **(A)**. Bars, 10 µm.

## Discussion

A previous report found the transgenerational fertility defect in LSD1 homolog *spr-5* and *spr-5;amx-1* double mutants, where double mutants of histone demethylases exacerbate fertility in *spr-5* mutants (Katz et al., 2009). Since all three belong to LSD1/2 histone demethylase homologs, it has been considered that they may perform similar functions. However, systematic analysis of how LSD2 homolog AMX-1*, per se*, contributes to defective fertility or sterility has not been investigated so far. Given that AMX-1 is requisite for proper fertility (Figure 2A), we examined how the lack of AMX-1 contributes to sterility in this study.

### AMX-1 Exhibits Distinct and Non-redundant Functions

It has been assumed that both AMX-1 and SPR-5 contribute to fertility defects since both are LSD1/2 histone demethylases. However, a few recent reports support their non-redundant functions. Specifically, while SPR-5 plays a role in double-strand break repair, AMX-1 functions in ICL repair (Nottke et al., 2009; Zhang et al., 2021). Second, AMX-1 and SPR-5 exhibit different tissue-specific expression profiles. For example, AMX-1 regulates the expression of H3K4me2 in the premeiotic tip, while SPR-5 does not. On the contrary, AMX-1 expression compensates for the lack of SPR-5 in the gut and embryonic cells, indicating their redundant functions as well (Zhang et al., 2021). Consistently, morphological changes in two histone demethylase mutants displayed both similarity and non-similarity. While both *amx-1* and *spr-5* sterile worms represented germline-less and spermless phenotypes, only *spr-5* mutants exhibited additional phenotypes, including chromatin bridges and packed embryos.

This study demonstrated that *amx-1* mutants reduced brood size over wild type throughout generations (Figure 1A). Also, unlike the progressive fertility defect observed in *spr-5* mutants, a reduction in *amx-1* was not significantly nor progressively altered between generations, indicating that AMX-1 is dispensable for transgenerational fertility defects, and phenotypes in two histone demethylases arise independently. Sterility exhibited in *amx-1* was not changed between early and late generations, supporting this idea as well (Figure 2A). Also, the SBS phenotype did not imprint on the following generations, further validating that both sterility and SBS in *amx-1* are transient and not transgenerational (Figure 2B).

### Sterility is Not the Outcome of Fertility Defects.

**FIGURE 8:**
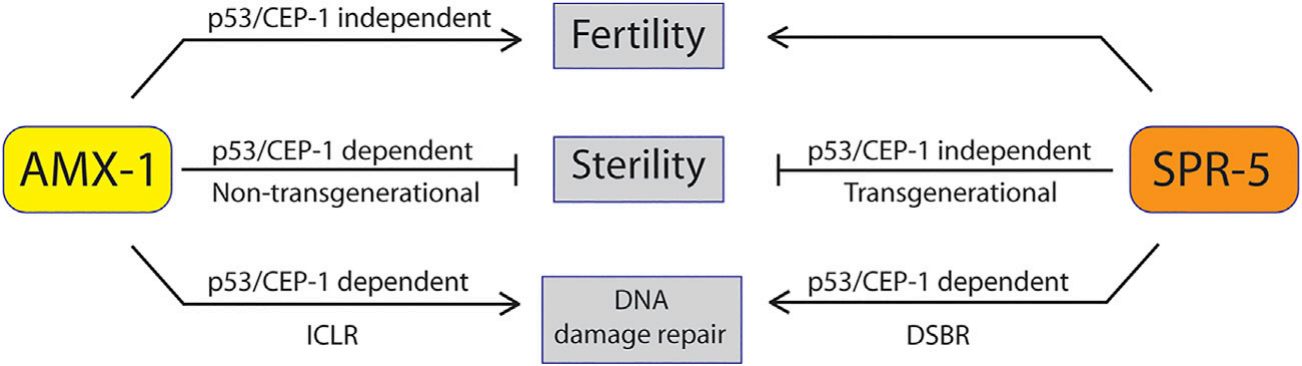
Model of AMX-1 in sterility compared with SPR-5. AMX-1 and SPR-5 function in both redundant and non-redundant manners. In wild-type animals, both AMX-1 and SPR-5 are necessary for proper fertility and sterility and maintenance of H3K4me2 level. While *spr-5* sterility does not require p53/CEP-1, *amx-1* sterility requires p53/CEP-1. AMX-1 modulates the adequate expression of Piwis in a CEP-1-dependent manner; thus, the absence of AMX-1 expression results in sterility. AMX-1 and SPR-5 are necessary for ICLR and DSBR, respectively.

Early generations of *spr-5* mutants exhibited a mild reduction of brood size. The decline became severer along with generations and often led to sterility (Katz et al., 2009), suggesting that sterility may have risen from the accumulation of severe fertility defects in the absence of SPR-5. Likewise, *amx-1* mutants displayed both sterility and fertility defects. However, unlike *spr-5*, sterility exhibited in *amx-1* is not the outcome of the progress fertility defects since defective fertility (SBS) resets in the following generations and is not transmitted to the subsequent generations. Furthermore, sterility rising regardless of generation supports this idea (Supplementary Figure S3). According to these observations, *amx-1* mutants displayed CEP-1/p53 dependent sterility (Figure 2A) and CEP-1 independent brood size reduction (Figure 1B), promoting the idea that sterility is not the consequence of reduced brood size. Therefore, defective fertility (brood size reduction) and sterility (zero fertility) originate from independent pathways in the absence of AMX-1 expression (Figure 8).

### AMX-1 Endows Fertility in a p53/CEP-1 Dependent Manner by Modulating Piwi Expression

Piwis are argonaute proteins associated with Piwi interaction small RNAs (26-31 nucleotides, piRNAs) rich in germ cells (Thomson and Lin, 2009; Juliano et al., 2011). Piwi’s functions include inhibition of the transposon and germline or stem cell renewal and lack of Piwi results in sterility in flies, Arabidopsis, and vertebrates. Several studies proposed that the sterility observed in the lack of piRNAs could be a consequence of transposition (Kidwell et al., 1977; Batista et al., 2008; Das et al., 2008). Plus, epigenetic contribution to sterility has also been proposed. *C. elegans* studies reported the connection between epigenetic regulation and Piwi’s function (Simon et al., 2014; Moore et al., 2019). Simon et al. (2014) *.* found that histone demethylase *spr-5* and *rbr-2* suppress sterility of *prg-1,* indicating that epigenetic regulation is necessary for proper Piwi functions. Interestingly, sterile *amx-1* and Piwi mutants share several phenotypes in common. First, both worms displayed disorganized and empty germlines and the number of sperms was reduced. Second, both mutants misregulated of Tc3 transposon expression, resulting from faulty Piwi expression. Also, worms displaying the sterility phenotype presented no obvious telomere fusion but showed normal six bivalent chromosomes allowing a reversible phenotype [Supplementary Figure S4, (Simon et al., 2014; Zhang et al., 2021)].

It is worth noting that while the lack of *prg-1* expression upregulates Tc3 expression (Das et al., 2008), a*mx-1* mutants display induced levels of Tc3 and Tc1 expression. Therefore, given that the lack of *amx-1* results in upregulation of both Piwis (*prg-1* and *prg-2*) and both transposons (Tc3 and Tc1), it is reasonable that the loss of AMX-1 result in broader impacts on biological pathways, not just restricted to defects in *prg-1*. Consistent with this idea, we reported that AMX-1 functions in spare roles in addition to the DNA damage-induced pathway (Zhang et al., 2021). Residual apoptosis found in *cep-1;amx-1* double mutants compared with *cep-1* single advocates the additional function of AMX-1. Also, we found that the increased Piwi and transposon expression in *amx-1* are partially suppressed by the *cep-1* mutant, whereas the sterility is fully suppressed by the *cep-1* mutant (Figures 6, 5 respectively). This suggests that moderate overexpression of transposable elements in *cep-1;amx-1* mutants might not be sufficient to induce sterility.

Given that sterility is not the consequence of global H3K4me2 accumulation in the germline, the H3K4me2 demethylase activity of AMX-1 in somatic tissues could be indispensable. A few reports support this idea. AMX-1 modulates the H3K4me2 level in germline and somatic cells (Wang et al., 2020; Zhang et al., 2021) and is required for proper mismatch repair MutL homolog expression (Zhang et al., 2021). Alternatively, histone demethylase independent activities might be necessary to regulate reproduction.

Likewise, the mammalian homolog of AMX-1 has been implicated in regulating gene transcription by modulating H3K4me2 (Fang et al., 2010). It is probable that AMX-1 may target both *prg-1* and *prg-2* based on the significant changes in mRNA expression levels (Figure 6). In line with this idea, a fly study demonstrates that histone demethylase LSD1 depletion results in Piwi-dependent desilencing of transposable elements. Moreover, LSD1 depletion leads to up-regulation of transposons (Lepesant et al., 2020). Thus, the lack of AMX-1 leads to dysregulation of Piwis, resulting in defective germline development and sterility (Figure 8). This idea may explain how *amx-1* exhibited upregulation of Tc3 since *prg-1* regulates the proper expression of Tc3 (Das et al., 2008). Alternatively, accumulation of H3K4me2 (which marks active chromatin status) in worms lacking AMX-1 expression may induce upregulation of Piwis followed by desilencing of Tc3. These two ideas may not be mutually exclusive. Identifying the targets for AMX-1 would help clarify AMX-1’s contribution to fertility and sterility. Together, our findings define a function for AMX-1 in promoting fertility in a p53/CEP-1 dependent manner by modulating Piwi expression.

## Data Availability

The original contributions presented in the study are included in the article/Supplementary Material, further inquiries can be directed to the corresponding author.
