# Supplementary material for: Histone Demethylase AMX-1 Regulates Fertility in a p53/CEP-1 Dependent Manner
Source: Front Genet. 2022 Jun 30;13:929716. doi: 10.3389/fgene.2022.929716 (PMC9280695; doi:10.3389/fgene.2022.929716)
Supplement: Supplementary file 1 [file DataSheet1.docx]

**
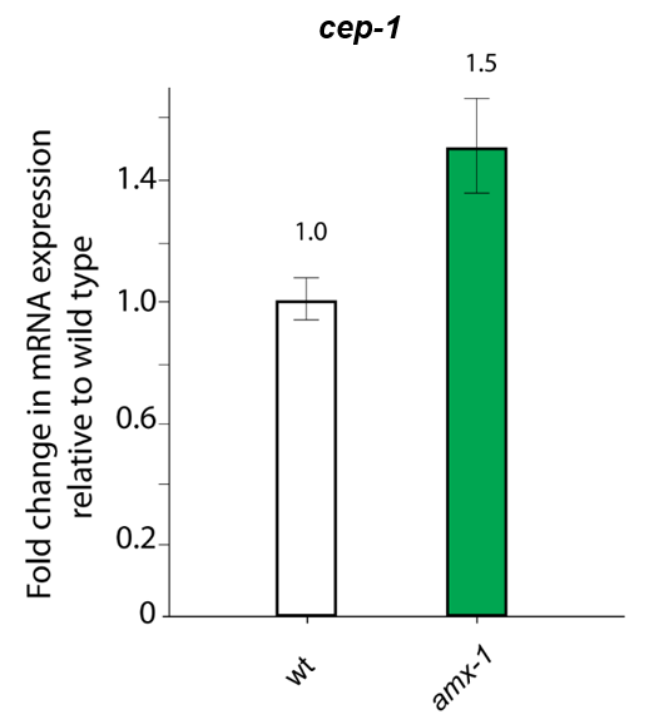
**

**Supplemental Figure 1**.

A lack of AMX-1 displayed a mild elevation of CEP-1 expression compared to wild type. A previous study found that *amx-1* mutants activate DNA damage response with intrinsic DNA damage, including the induced level of phospho-CHK-1 and DNA damage apoptosis in CEP-1 dependent manner [15]. In accordance with these results, a mild induction of CEP-1 was observed.


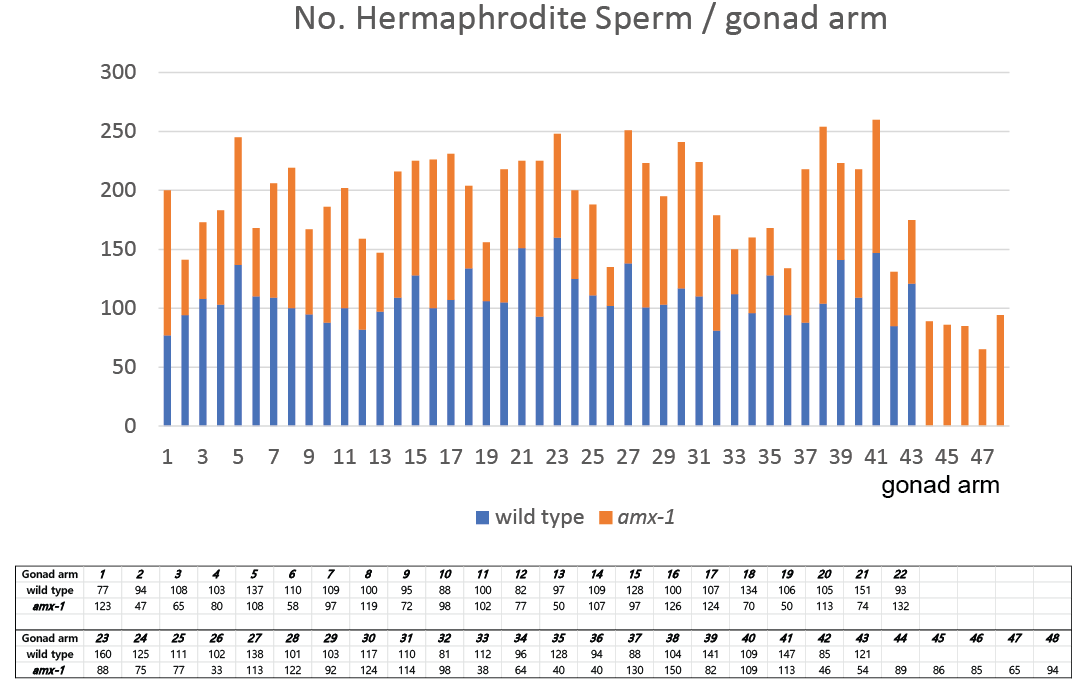


**Supplemental Figure 2**.

The number of sperms was counted in one gonad arm of each worm. 20-hour post-L4 animals were stained with DAPI without dissection. Raw data for Figure 4C.


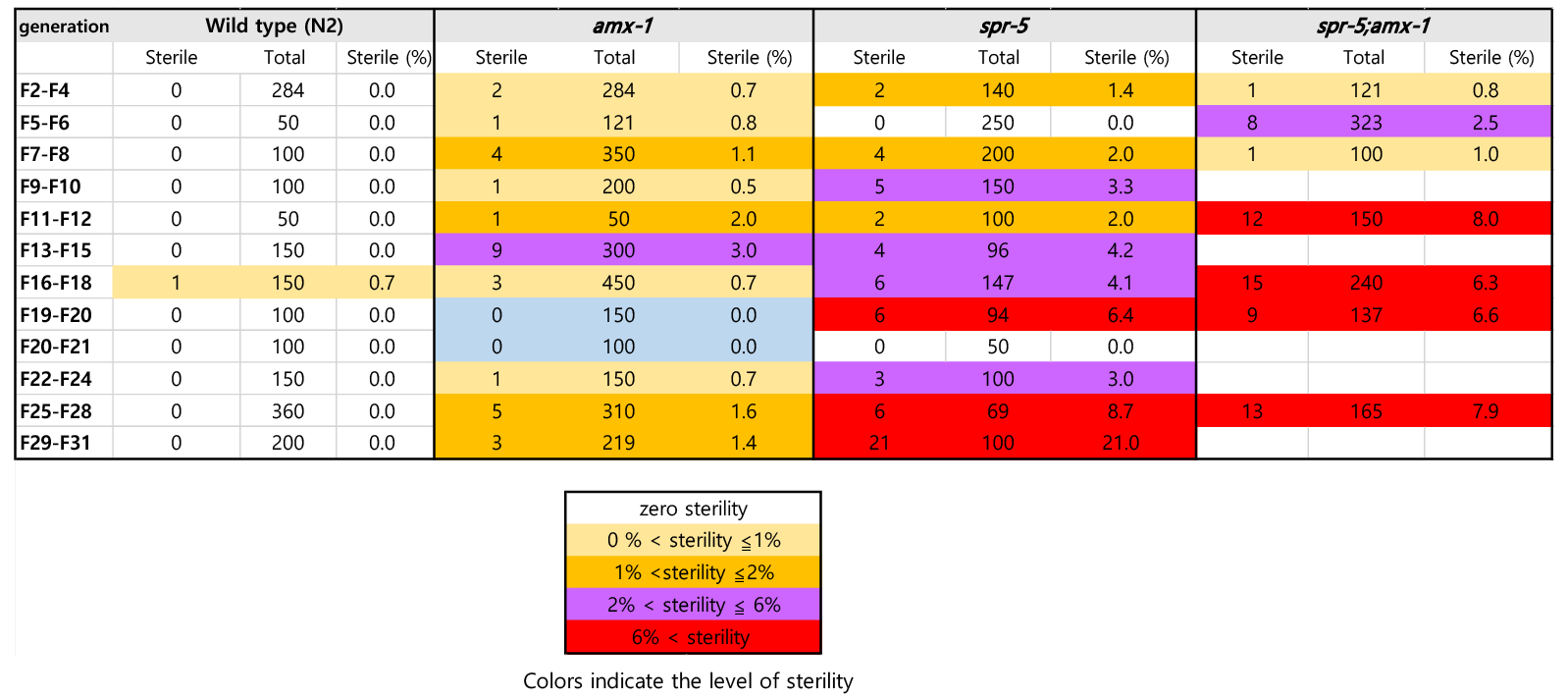


**Supplemental Figure 3**.

**Sterility was scored when worms produced no viable progenies until two days post L4 stage.** Sterility was observed regardless of generations (F2-F31). Sterility in *amx-1* occurs regardless of generations, while sterility in *spr-5* is more frequent in later generations. Colors indicate the level of sterility.


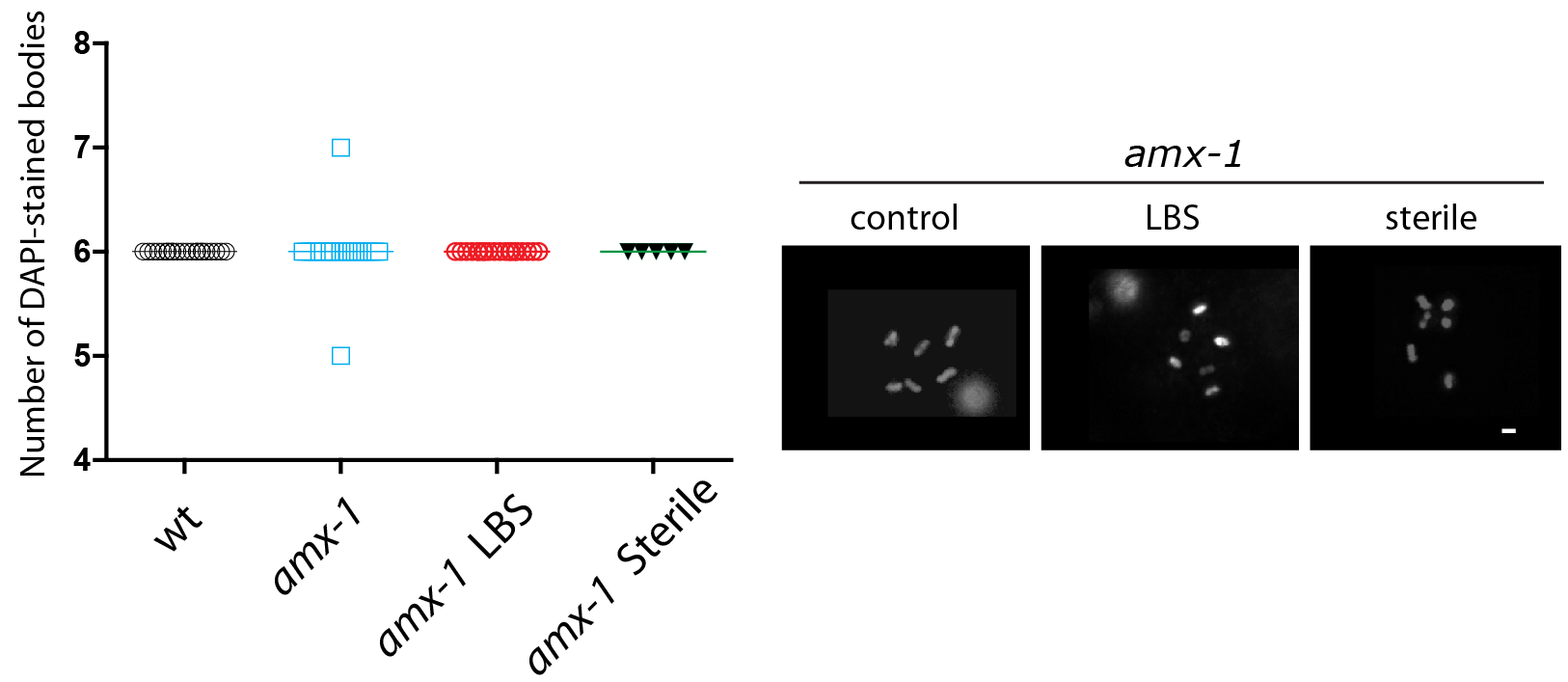


**Supplemental Figure 4**.

**The number of DAPI-stained bodies was observed in diakinesis oocytes.** Worms displaying sterility presented no telomere fusion but showed normal six bivalent chromosomes. The number of -1 or -2 oocytes scored. N=20-25 worms except for sterile worms (n=5). Since some standard deviations are zero, it is impossible to perform statistical analysis. Bars, 2 µm.

**Supplemental Table 1**. **Primer information for gene expression assessed by qPCR.**

| **Gene** | **Description** | **Sequence** | **Forward primer** | **Reverse primer** |
| --- | --- | --- | --- | --- |
| ***prg-1*** | piwi like RNA-mediated gene silencing 1 | D2030.6 | TGTTCCCACTCCTCCTTCAG | ATGTTTCGCCAACTTCATCC |
|  |  |  |  |  |
| ***prg-2*** | piwi like RNA-mediated gene silencing 2 | C01G5.2 | CGTATCCGACGATCATTGTG | CAATCAATGCGTGAATTTGC |
|  |  |  |  |  |
| ***Tc1*** | Transposable element |  | CACATGACGACGTTGAAACC | AACCGTTAAGCATGGAGGTG |
|  |  |  |  |  |
| ***Tc3*** | Transposable element |  | AATAGTCGCGGGTTGAGTTG | GAGCGTTCACGGAGAAGAAG |
|  |  |  |  |  |
| ***cep-1*** | *Caenorhabditis elegans* p53 homolog | F52B5.5 | TTCCGACGCAAGTAGTCTCC | CCGTTTGCATTGAACAACAC |
